# Syntaxin 1B Mediates Berberine’s Roles in Epilepsy-Like Behavior in a Pentylenetetrazole-Induced Seizure Zebrafish Model

**DOI:** 10.3389/fnmol.2018.00378

**Published:** 2018-11-26

**Authors:** Yang-Min Zheng, Bo Chen, Jian-Dong Jiang, Jing-Pu Zhang

**Affiliations:** ^1^NHC Key Laboratory of Biotechnology of Antibiotics, Institute of Medicinal Biotechnology, Chinese Academy of Medical Sciences & Peking Union Medical College, Beijing, China; ^2^Beijing Key Laboratory of Antimicrobial Agents, Institute of Medicinal Biotechnology, Chinese Academy of Medical Sciences & Peking Union Medical College, Beijing, China; ^3^State Key Laboratory of Bioactive Substances and Functions of Natural Medicines, Institute of Materia Medica, Chinese Academy of Medical Sciences & Peking Union Medical College, Beijing, China

**Keywords:** STX1B, berberine, epilepsy, photosensitive seizure, PTZ, zebrafish

## Abstract

Epilepsy is a neuronal dysfunction syndrome characterized by transient and diffusely abnormal discharges of neurons in the brain. Previous studies have shown that mutations in the *syntaxin 1b* (*stx1b*) gene cause a familial, fever-associated epilepsy syndrome. It is unclear as to whether the *stx1b* gene also correlates with other stimulations such as flashing and/or mediates the effects of antiepileptic drugs. In this study, we found that the expression of *stx1b* was present mainly in the brain and was negatively correlated with seizures in a pentylenetetrazole (PTZ)-induced seizure zebrafish model. The transcription of *stx1b* was inhibited by PTZ but rescued by valproate, a broad-spectrum epilepsy treatment drug. In the PTZ–seizure zebrafish model, *stx1b* knockdown aggravated larvae hyperexcitatory swimming and prompted abnormal trajectory movements, particularly under lighting stimulation; at the same time, the expression levels of the neuronal activity marker gene *c-fos* increased significantly in the brain. In contrast, *stx1b* overexpression attenuated seizures and decreased *c-fos* expression levels following PTZ-induced seizures in larvae. Thus, we speculated that a deficiency of *stx1b* gene expression may be related with the onset occurrence of clinical seizures, particularly photosensitive seizures. In addition, we found that berberine (BBR) reduced larvae hyperexcitatory locomotion and abnormal movement trajectory in a concentration-dependent manner, slowed down excessive photosensitive seizure-like swimming, and assisted in the recovery of the expression levels of STX1B. Under the downregulation of STX1B, BBR’s roles were limited: specifically, it only slightly regulated the levels of the two genes *stx1b* and *c-fos* and the hyperexcitatory motion of zebrafish in dark conditions and had no effect on the overexcited swimming behavior seen in conjunction with lighting stimulation. These findings further demonstrate that STX1B protein levels are negatively correlated with a seizure and can decrease the sensitivity of the photosensitive response in a PTZ-induced seizure zebrafish larvae; furthermore, STX1B may partially mediate the anticonvulsant effect of BBR. Additional investigation regarding the relationship between STX1B, BBR, and seizures could provide new cues for the development of novel anticonvulsant drugs.

## Introduction

Epilepsy is a chronic neurological disease with a high prevalence characterized by spontaneous seizures, abnormal discharges of the brain, and convulsion. According to statistics, 1% of the global population suffers from epilepsy; among them, children, 1 out of 200 of whom are affected (Cowan, 2002; Poduri and Lowenstein, 2011). According to the International League Against Epilepsy 2017 Classification of Seizure Types Basic Version, three major types exist – focal onset, generalized onset, and unknown onset – in which the motor type of seizure is involved. Notably, hyperkinetic seizures have been specified as a subtype of motor onset under focal onset. Patients with motor onset usually suffer a sudden loss of consciousness and symptoms such as rigidity and convulsion. Furthermore, about 20% of epilepsy patients demonstrate other mental illnesses due to anxiety and sleep problems (Sillanpaa et al., 2016; Besag, 2018). Therefore, epilepsy is a serious social burden and a threat to patients in terms of both their physical and mental health, and often brings about great loss of property.

Photosensitive epilepsy is caused by visual stimuli with an abnormal electroencephalogram response, which is known as a photoparoxysmal response (Fisher et al., 2005). Recently, people are increasingly coming into contact with more electronic devices, such as televisions, computers, cameras, and other similar items. Unfortunately, this growth in intermittent photic stimulation has greatly increased the prevalence of epileptic seizures. Therefore, the incidence of photosensitive epilepsy is also increasing (Poleon and Szaflarski, 2017), with approximately 5% of epilepsy patients being affected (Martins da Silva and Leal, 2017). A recent study employed gene sequencing to identify the cause of the archetypal generalized photosensitive epilepsy syndrome as a chromodomain helicase DNA-binding protein 2 (CHD2) mutation and found approximately five times as many CHD2 variants in photosensitive epilepsy patients as in the controls (Galizia et al., 2015; Poleon and Szaflarski, 2017). According to another study, bromodomain-containing protein 2 might be an underlying susceptible gene for the photoparoxysmal response (Lorenz et al., 2006). However, despite the efforts of these investigations, the pathogenesis of photosensitive epilepsy is still unclear.

Syntaxin 1b (STX1B) is a soluble, N-ethylmaleimide-sensitive fusion attachment receptor (SNARE) protein located in the presynaptic membrane that mediates the fusion of the synapse vesicle and the target membrane, promotes the release of neurotransmitters, and is expressed in the central nervous system (Sollner et al., 1993; Sudhof, 2013; Zhou et al., 2013). According to previous reports, the mutation of *stx1b* is related to the onset of familial fever-associated epilepsy syndromes. In previous research, *stx1b* knockdown presented abnormal electrographic activity in zebrafish larvae under hyperthermic conditions (Schubert et al., 2014; Kearney, 2015). Clinical observation found that the presentation of myoclonic astatic epilepsy (MAE) was also related to the variant or deletion of the *stx1b* gene, suggesting that STX1B should closely observed in the diagnosis of MAE (Vlaskamp et al., 2016). Whether STX1B is involved in photosensitive epilepsy or not has to our knowledge, not yet been reported on.

Berberine (BBR) is a natural compound extracted from the traditional Chinese herb *Coptis* *chinensis* and has for many years been known to have a good effect on diarrhea. Studies have shown that BBR also has potential therapeutic effects in diabetes (Zhang et al., 2010), hyperlipidemia (Kong et al., 2004; Kim et al., 2009), heart disease (Lau et al., 2001; Zeng et al., 2003), and inflammation (Choi et al., 2006; Lou et al., 2011). In addition, BBR was found to have a neuroprotective effect on multiple central nervous system diseases, such as Alzheimer’s disease and epilepsy (Kulkarni and Dhir, 2010; Gao et al., 2014; Hussien et al., 2018). In one study, BBR notably improved cognitive behavior in a rat model of Alzheimer’s disease and inhibited the formation of Aβ42, a main constituent of amyloid-β plaques associated with the neurodegenerative condition (Hussien et al., 2018). Another investigation reported that BBR increased the levels of both interleukin 1β and inducible nitric oxide synthase to mediate neuroprotective properties and ameliorated spatial memory impairment in a rat model of Alzheimer’s disease (Zhu and Qian, 2006). In a kainate-induced temporal lobe seizure rat model, BBR significantly decreased the incidence of seizures (Mojarad and Roghani, 2014). Furthermore, in a pilocarpine-induced seizure rat model, BBR delayed both latency to the first seizure and time to the development of status epilepticus (Gao et al., 2014). However, few studies on the antiepileptic mechanism of BBR have been published to date. Pentylenetetrazole (PTZ) is a gamma-aminobutyric acid (GABA) receptor inhibitor (Macdonald and Barker, 1977) capable of resisting the inhibitory effect of GABA on neural activity and is often used in seizure models in rodents and zebrafish (Baraban et al., 2005; Stewart et al., 2012; Epps and Weinshenker, 2013; Grone and Baraban, 2015). A number of studies have presented zebrafish epilepsy-like seizures via PTZ induction models over the past 10 years (Baraban et al., 2005; Ellis and Soanes, 2012; Stewart et al., 2012; Gupta et al., 2014; Rahn et al., 2014; Torres-Hernandez et al., 2015; Barbalho et al., 2016). Referring to Barabans’ research (Baraban et al., 2005), we established a zebrafish seizure model using PTZ and studied the zebrafish convulsive episodes under a dark condition and lighting stimulation; using this model, the correlations of STX1B with seizures and the anticonvulsant effects of BBR were investigated. We found that BBR can promote the expression of STX1B directly or indirectly and alleviate epilepsy-like seizures, especially photosensitive seizures in PTZ-induced seizure zebrafish larvae.

## Materials and Methods

### Zebrafish Feeding and Care

AB wild-type line zebrafish (*Danio rerio*) were obtained from the College of Life Sciences and Technology of Tsinghua University in Beijing, China. The zebrafish were raised under standard laboratory conditions with a 14-h light/10-h dark cycle at a temperature of 28.5°C ± 1°C (Kimmel et al., 1995). Zebrafish embryos and larvae were incubated in the rearing water of 280 mg/L Tropical Marine Artificial Seawater Crystal (CNSIC Marine Biotechnology Co., Ltd., Tianjin, China), with a conductivity of 450 to 550 μS. This research was reviewed and approved by the Laboratory Animal Management and Animal Welfare Committee at the Institute of Medicinal Biotechnology of the Chinese Academy of Medical Sciences. The zebrafish experimental protocols complied with the Ethics of Animal Experiments guidelines set by the Institute of Medicinal Biotechnology of the Chinese Academy of Medical Sciences.

### Microinjection

Two *stx1b* morpholino oligos and a scrambled morpholino oligo were purchased from Gene Tools, LLC (Philomath, OR, United States). The two *stx1b* morpholino oligo sequences were as follows: 5′-GTGCGATCCTTCATTTTTCCCCGCC-3′ (*stx1b*-MO1) and 5′-AAATATCTCTTGAGATGTCCGCTGC-3′ (*stx1b*-MO2) (Schubert et al., 2014), which are the *stx1b* antisense oligos used to inhibit STX1B expression by binding to STX1B initiation codon sites. The scrambled morpholino oligo with a randomized 25-base sequence designed by Gene Tools^1^ (Philomath, OR, United States) was used as a nonsense control for *stx1b*-MO. As part of the present study, 0.5 nL of 50 μM *stx1b*-MO1 or *stx1b*-MO2 was injected into each embryo of the 1–4-cell stage, and the embryos were subsequently cultivated in the rearing water as described above. STX1B overexpression was prompted via injection of 0.5 nL of pIRES2-*stx1b*-EGFP and pIRES2-EGFP (as a mock control) at a concentration of 60 ng/μL. The injected embryos at 5 days postfertilization (dpf) were collected for subsequent experiments.

### Chemical Treatment

Berberine was obtained from the National Institutes for Food and Drug Control (Beijing, China). Valproate (VPA) (valproic acid sodium salt, P4543) and PTZ (P6500) were purchased from Sigma-Aldrich (St. Louis, MO, United States).

For the seizure model group, we essentially followed the method described by Baraban et al. (2005). Briefly, zebrafish larvae at 7 dpf were exposed to a PTZ solution at concentrations of 2, 4, and 6 mM, respectively, for 1 h and then collected for a behavioral experiment or for 2 h and then collected for *in situ* hybridization and western blotting experiments. Based on the results of the PTZ dose experiment, 4 mM of PTZ was used for the subsequent experiments conducted in the PTZ–seizure-related groups. Each group contained 24 larvae.

For the drug-treated groups, wild-type larvae and injected larvae at 5 dpf were exposed to BBR at concentrations of 25, 50, and 75 μM or to VPA at concentrations of 60, 120, and 240 μM for 2 days (7 dpf), respectively, after being washed three times with the normal rearing solution. Then, the larvae were exposed to 4 mM of PTZ for 1 h and collected for a behavioral experiment, or after 2 h collected for subsequent experiments including whole-mount *in situ* hybridization and western blotting detections for *c-fos* and *stx1b* transcription and protein levels.

### Whole-Mount *in situ* Hybridization

Sense and antisense RNA probes of the genes *c-fos* and *stx1b* were synthesized using a digoxigenin RNA labeling kit (1175025; Roche Applied Science, Penzberg, Germany) and complementary DNA fragment templates that were amplified using reverse transcription-polymerase chain reaction and inserted into a pGEM-T plasmid. Gene *c-fos* primer pair sequences were as follows: 5′-AACTGTCACGGCGATCTCTT-3′ (the forward primer) and 5′-CTTGCAGATGGGTTTGTGTG (the reverse primer) (Baraban et al., 2005). Gene *stx1b* primer pair sequences were as follows: 5′-GCAGCACCAAACCCTGATGAAA (the forward primer) and 5′-CCTCCGATACTGGACCGCAAAA (the reverse primer). Larvae were fixed with 4% paraformaldehyde overnight at 4°C before being stored in methanol at 4°C. Procedures for whole-mount *in situ* hybridization were performed as described by Whitlock and Westerfield (2000).

### Western Blotting

For western blot analysis, total proteins were extracted from zebrafish larvae with a RIPA lysis kit (C1053; Applygen Technologies Inc., Beijing, China), separated using 12% sodium dodecyl sulfate polyacrylamide gel electrophoresis, and transferred to a nitrocellulose filter (T41524; PALL, Mexico). Protein blots were blocked with 5% milk in Tris-buffered saline for 1 h at room temperature, with antibodies against STX1B (1:1,000 dilution; 110 403; Synaptic Systems, Coventry, United Kingdom) and β-actin (1:2,000 dilution; A5441; Sigma-Aldrich, St. Louis, MO, United States). The blots were incubated with secondary antibodies (goat anti-mouse or goat anti-rabbit immunoglobulin G from ZSGB-BIO, Beijing, China) for 1 h and visualized by an immobilon western chemiluminescent horseradish peroxidase substrate (Millipore, Billerica, MA, United States). The western blotting was performed in parallel three times.

### Behavioral Experiment

All of the zebrafish swimming activity was analyzed at 7 dpf by the ZebraLab Video-Track system version 3.3 (ViewPoint Life Science, Montreal, QC, Canada). The zebrafish larvae were individually placed into the wells of a 48-well plate (1 fish/well). Locomotor distance, velocity, and swimming tracks were separately recorded in two kinds of conditions. In the first, the larvae stayed in a dark box and their swimming actions were recorded for 20 min, during which time the data and moving tracks were collected once every 2 min, with a red trajectory indicating an abnormal swimming trajectory and an overspeed higher than 4 cm/s defined as a highly active movement and a green trajectory indicating a velocity between 0.2 and 4 cm/s defined as an active movement, respectively. The second condition involved a shift experiment between dark and light, in which the zebrafish larvae were subjected to three cycles of 5 min dark and 10 s light periods, with data collected once every 10 s.

**FIGURE 1:**
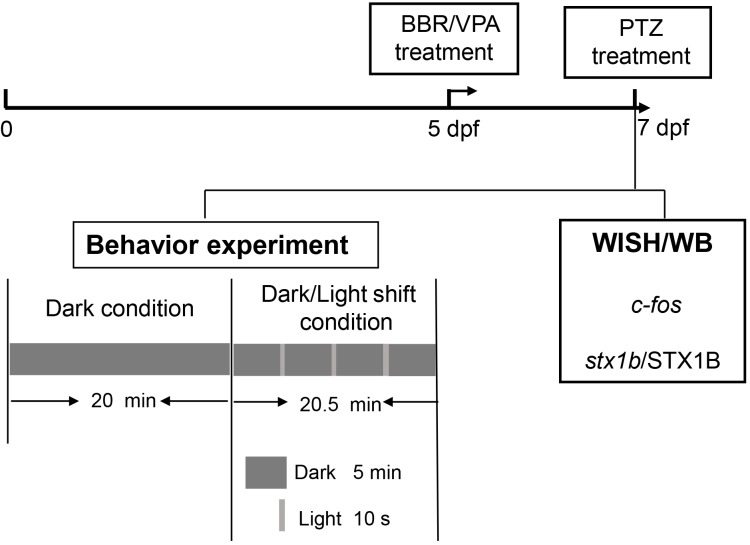
A representative flowchart of the experimental procedure and methods in this study.

The experimental procedure and pharmacological manipulations in this study are depicted in the flowchart in Figure 1.

### Statistical Analysis

All data were plotted by using GraphPad Prism 5.0 (GraphPad Software Inc., La Jolla, CA, United States). Comparisons between multiple groups were carried out using the analysis of variance test. Significance for all tests was defined at ^∗/#/&/Φ/𝜃/φ^^/§^ *P* < 0.05; ^∗∗/##/&&/ΦΦ/𝜃𝜃/φ^ ^φ^^/§§^ *P* < 0.01; and ^∗∗∗/###/&&&/ΦΦΦ/𝜃𝜃𝜃/φ^ ^φ^ ^φ^^/§§§^ *P* < 0.001.

## Results

### PTZ Induced a Zebrafish Epilepsy-Like Seizure Model and Suppressed Expression of *stx1b* Gene in Zebrafish Larvae Brains

**FIGURE 2:**
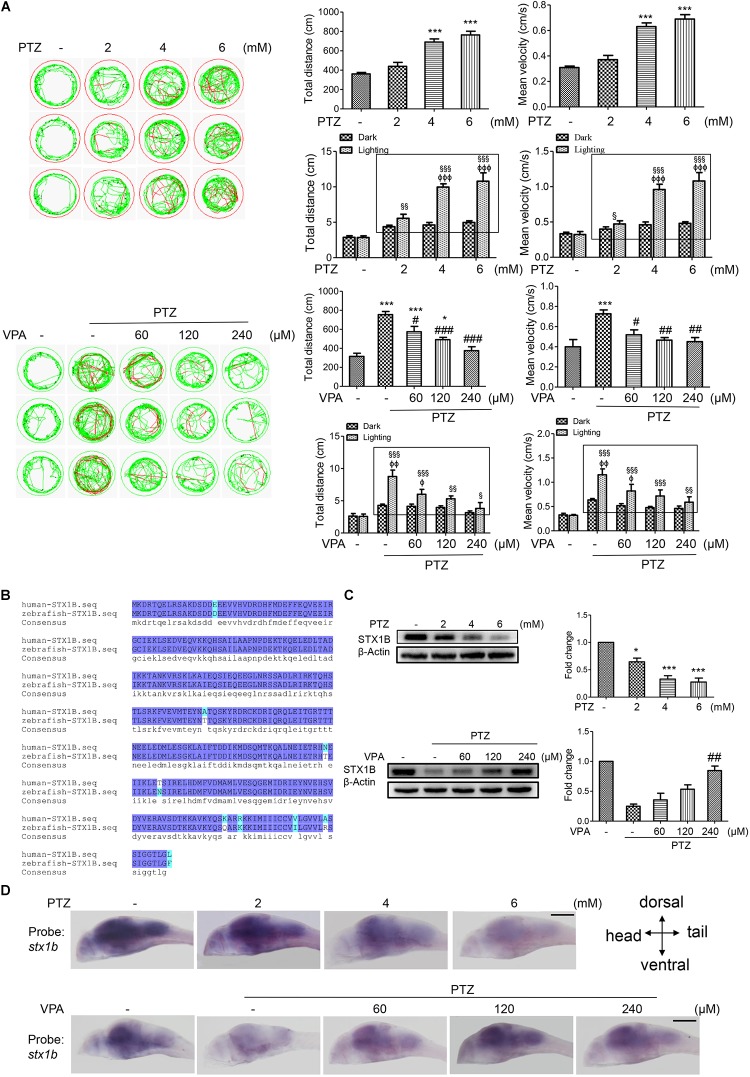
*Stx1b* gene expression was suppressed in PTZ-induced seizure zebrafish larvae. **(A)** Seizure-like swimming was induced by PTZ in zebrafish larvae. The left track panel and the right upper histograms show the larval swimming behavior (distance and speed) during 20 min in the dark condition. The red trajectory indicates overactive movement and the green trajectory indicates active movement in the left track figure. The right upper histograms show the larval swimming distance and speed, which were recorded for 20 min in the dark condition. The right lower histograms show the larval swimming distance and speed in three cycles of 5 min dark and 10 s light periods; the open boxes show the differences in distance and velocity of the overexcited larvae between the dark and light conditions (*n* = 24). **(B)** Alignment of human and zebrafish STX1B amino acid sequences. The amino acids shown in dark blue are identical, those in shallow blue demonstrate amino acids with similar polarity, and those in white/shallow blue are different. **(C)** Western blotting tests indicated that STX1B protein was decreased by PTZ (Upper) and increased by VPA (Lower) in a concentration-dependent manner (*n* = 3). ^∗^*P* < 0.05 and ^∗∗∗^*P* < 0.001 vs. wild-type; *^#^P* < 0.05, ^##^*P* < 0.01, and ^###^*P* < 0.001 vs. PTZ model; ^§^ *P* < 0.05, ^§§^ *P* < 0.01, and ^§§§^*P* < 0.001 vs. wild-type in the light condition; ^Φ^*P* < 0.01, ^ΦΦ^*P* < 0.01 and ^ΦΦΦ^*P* < 0.001 indicated light vs. dark in the same set of conditions. **(D)** Hybridization *in situ* results show STX1B gene expression in the larval brain inhibited by PTZ and rescued by VPA in a concentration-dependent manner (*n* = 20).

Previous research has reported that the human *stx1b* gene is associated with familial fever-associated epilepsy syndromes and plays a part in rescuing the function of *stx1b* knockdown in zebrafish (Schubert et al., 2014; Kearney, 2015). In the present study, we are interested in whether STX1B is also related to the seizures caused by PTZ and if it mediates the effects of the antiepileptic drugs VPA and BBR in zebrafish. First, we set up a zebrafish seizure model using PTZ and confirmed the model by use of a VPA. In this model, larvae swimming distance, velocity, and abnormal trajectory were significantly increased in a PTZ dose-dependent manner and were aggravated particularly under the condition of a shift between dark and light (Figure 2A). VPA showed an obvious therapeutic effect on the seizure-like swimming; specifically, the PTZ-induced larval overspeed swimming was slowed down in a VPA dose-dependent manner under both the dark condition and the dark–light shift condition (Figure 2A). Then, we compared the homology between human and zebrafish STX1B protein sequences. Each of these two STX1B proteins consist of 288 amino acids with a positive ratio of 98% and an identity ratio of 96.8%, in which only 5 amino acids are different and 4 amino acids have similar polarity (Figure 2B). Therefore, it can be speculated that both proteins may have similar biological functions. Western blotting confirmed that the STX1B protein was decreased by PTZ and increased by VPA in larvae (Figure 2C). In addition, hybridization *in situ* results showed that the *stx1b* gene was expressed mainly in the brain region and clearly downregulated by PTZ and recovered by VPA in a dose-dependent manner (Figure 2D). This overexcited behavior was inversely proportional to the STX1B level. These results indicate that the STX1B level is negatively associated with PTZ-induced seizures in zebrafish and more closely correlated with a photosensitive seizure. Based on these results, we chose a PTZ concentration of 4 mM for our PTZ-induced seizure model and a VPA dose of 120 μM as a positive control in the following experiments.

### Level of *stx1b* Correlates Inversely With PTZ-Induced Seizure in Zebrafish Larvae

**FIGURE 3:**
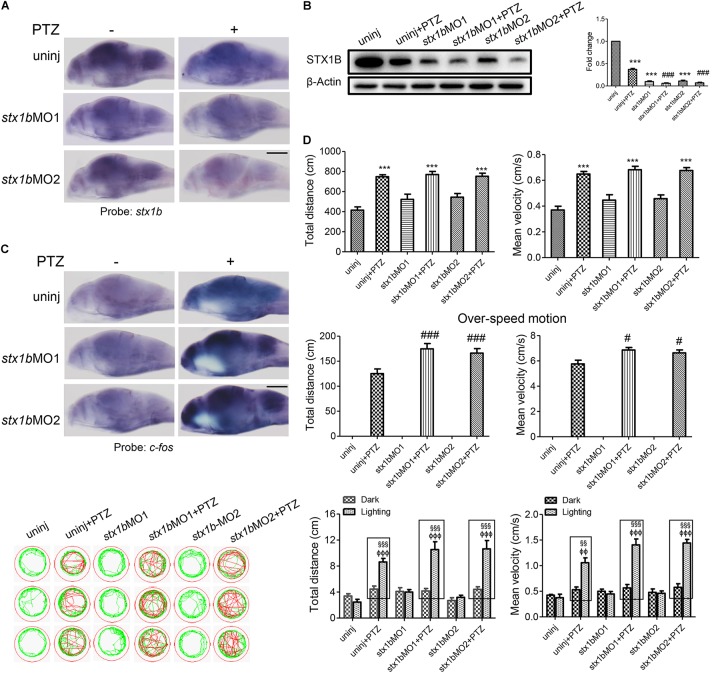
Larval seizure was aggravated by downregulation of *stx1b* transcription in the PTZ-induced seizure model. Levels of *stx1b* gene transcription (*n* = 20) **(A)** and STX1B protein (*n* = 3) **(B)** were reduced and *c-fos* gene transcription was increased in the larval (7 dpf) brain (*n* = 20) **(C)** by *stx1b* morpholino oligos injection in the PTZ model, as compared with in the PTZ-only and the morpholino oligos-only injection models. The larval swimming experiment (*n* = 24) **(D)** showed that average speed and total distance were not changed, but that the abnormal pathway and overspeed were increased following 20 min in the dark condition and that photosensitive seizure was aggravated under the condition of light–dark transition with 5 min in the dark and 10 s in the light for three cycles in the PTZ plus *stx1b* morpholino oligos larvae, as compared with the two groups of the PTZ-only and the *stx1b* morpholino oligos-only injection models. The data show average speeds during the 20 min in the dark and the 10 s in the dark–light transformation; the boxes indicate the difference of locomotion distances and speeds between the light–dark transitions. Swimming tracks were recorded at 2 min in the dark condition and the red trajectory indicates overactive movement and the green trajectory indicates active movement. *stx1b*-MO1 and *stx1b*-MO2 were two morpholino oligos that bound to the *stx1b* messenger RNA initiate sequence with a different sequence; by using two target oligos, their inhibition effect was confirmed with each other. ^∗∗∗^*P* < 0.001 vs. wild-type; ^#^*P* < 0.05 and ^###^*P* < 0.001 vs. PTZ model; ^§§^*P* < 0.01 and ^§§§^*P* < 0.001 vs. wild-type in the light condition; ^ΦΦ^*P* < 0.01 and ^ΦΦΦ^*P* < 0.001 indicates light vs. dark.

Further, we investigated whether STX1B could affect PTZ-induced seizures, especially under lighting stimulation using gene knockdown and overexpression methods. A *stx1b* overexpression plasmid and two *stx1b* morpholino oligos were separately injected into zebrafish embryos to upregulate or downregulate *stx1b* gene expression. When the zebrafish embryos injected with the *stx1b* morpholino oligos were exposed to PTZ, *stx1b* messenger RNA and protein levels were lower (Figures 3A,B) and the neuronal activity marker *c-fos* level was higher (Figure 3C) than in those larvae exposed only to PTZ or that received only a morpholino oligos injection. This suggests that the downregulation of STX1B combined with PTZ exposure worsened dysregulation of the two genes’ expression. Additionally, behavioral experiments showed that the knockdown of *stx1b* aggravated the abnormal swimming pathway and velocity instead of the total average velocity and distance in the dark condition and also intensified overexcited behavior under light stimulation induced by PTZ, in comparison with in the PTZ-only and morpholino oligos injection-only groups (Figure 3D). The behavior changes between the wild-type group and the groups that underwent morpholino oligos injection without PTZ induction were minor or not observed, meaning that the existence of a partial deficiency of STX1B in wild-type larvae did not affect their behavior too significantly. A scrambled MO as a nonsense control for *stx1b*-MO showed no effects on the expression of *stx1b* and *c-fos* and also did not change larval swimming behavior in comparison with the uninjected and PTZ-induction groups (relevant data supplied in the Supplementary Material). These results imply that the downregulation of STX1B probably promoted the onset of epilepsy-like seizures, particularly in the case of photic stimulation on the PTZ-treated zebrafish.

**FIGURE 4:**
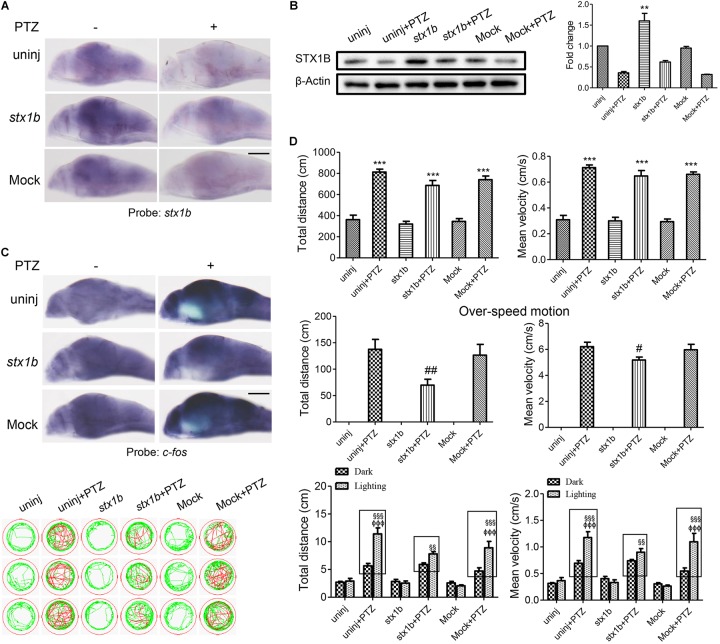
Larval seizure-like behavior was reduced by increased STX1B level in a PTZ-induced seizure model. **(A)** Both *stx1b* transcription in the wild-type and PTZ models were enhanced in larval (7 dpf) brains by *stx1b* injection as compared with that following no injection and mock injection (*n* = 20). **(B)** Western blotting confirmed differential levels of STX1B protein among the variant groups; notably, the STX1B level was raised in the *stx1b*-PTZ group as compared with in the PTZ-only group (*n* = 3). **(C)** *c-fos* messenger RNA was decreased by STX1B overexpression in the *stx1b*-PTZ model versus in the PTZ model group or the mock-PTZ group (*n* = 20). **(D)** The larval swimming experiment showed that, with STX1B overexpression, average speed and total distance were not obviously changed but abnormal pathway and overspeed were significantly decreased with 20 min in the dark condition, while photosensitive seizure was inhibited under the condition of light–dark shift in the PTZ-model larvae as compared with in the two groups of the PTZ-only model and the PTZ plus mock injection model (*n* = 24). The rectangles indicate differential responses between light–dark transitions in the three groups of the PTZ-model larvae. ^∗∗^*P* < 0.01 and ^∗∗∗^*P* < 0.001 vs. wild-type; ^#^*P* < 0.05 and ^##^*P* < 0.01 vs. PTZ model; ^§§^ *P* < 0.01 and ^§§§^ *P* < 0.001 vs. wild-type in the light condition; ^ΦΦΦ^*P* < 0.001 indicates light vs. dark.

To further verify these outcomes, we constructed a *stx1b* overexpression vector and injected it into zebrafish embryos. As shown in Figures 4A,B, in the group of PTZ plus *stx1b* overexpression, the levels of *stx1b* messenger RNA and protein were higher than in the PTZ-treated group; in addition the expression of *c-fos* in the brain was significantly lower than in the PTZ-treated group (Figure 4C). Behavioral experiment results showed that the overexpression of STX1B had no significant effect on the total distance and average velocity of the PTZ-injected zebrafish, but had a notable reducing effect on abnormal trajectory and overspeed locomotion in the dark condition (Figure 4D). Moreover, the overexpression of STX1B significantly slowed down the PTZ-induced larval overexcited response in the dark–light shift condition (Figure 4D). Those results confirm that the upregulation of STX1B alleviated the seizure, including in particular a photosensitive seizure, in PTZ-treated zebrafish, suggesting that the overexpression of STX1B might have a potential protective effect in a PTZ-induced seizure model.

### Berberine Reduced the PTZ-Induced Seizure-Like Response by Promoting *stx1b* Gene Expression

**FIGURE 5:**
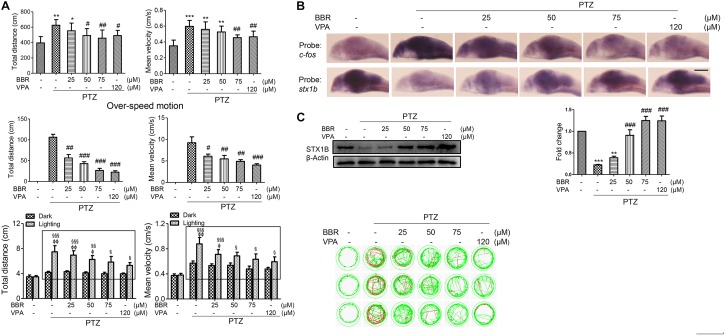
Berberine (BBR) inhibited seizure in PTZ-model zebrafish with the increase of STX1B level. **(A)** A behavioral experiment showed that BBR inhibited the larval overexcited locomotion in speed and distance under the conditions of non-stimulation and dark–light cycling stimulation in PTZ-model larvae. A representative swimming trajectory (2 min) is presented (*n* = 24). The rectangles showed differential distances and speeds between light–dark transitions in the groups of PTZ plus BBR larvae as compared with in the PTZ-only model. **(B)** Hybridization *in situ* showed that BBR inhibited the increase of *c-fos* and rescued *stx1b* descending induced by PTZ in the larval (7 dpf) brains (*n* = 20). **(C)** Western blotting results confirmed that BBR recovered STX1B protein levels to almost normal in a dose-dependent manner (*n* = 3). ^∗^*P* < 0.05, ^∗∗^*P* < 0.01, and ^∗∗∗^*P* < 0.001 vs. wild-type; ^#^*P* < 0.05, ^##^*P* < 0.01, and ^###^*P* < 0.001 vs. PTZ model; ^§^ *P* < 0.05, ^§§^ *P* < 0.01, and ^§§§^ *P* < 0.001 vs. wild-type in the light condition; ^Φ^*P* < 0.01 and ^ΦΦ^*P* < 0.01 indicates light vs. dark.

Previous studies have reported that the use of BBR significantly decreased the incidence of seizures in a seizure rat model (Mojarad and Roghani, 2014) and delayed both latency to the first seizure and time to develop status epilepticus in a pilocarpine-induced seizure rat model (Gao et al., 2014). However, few studies on the anticonvulsant mechanism of BBR have been reported at this time. In this work, we are interested in researching whether the *stx1b* gene correlates with the BBR anticonvulsant effect. A larval swimming experiment was first performed and the results showed that BBR reduced larval average velocity and total movement distance including abnormal swimming track and overspeed in the dark condition; in addition, BBR also more obviously alleviated a PTZ-induced overexcited response in the light stimulation condition in PTZ-induced zebrafish, in a dose-dependent manner (Figure 5A). *In situ* hybridization results showed that BBR inhibited the increase of the *c-fos* level induced by PTZ and promoted STX1B expression in a concentration-dependent manner in the brain of PTZ-treated larvae (Figure 5B). Furthermore, a western blotting test also confirmed that STX1B protein increased in a BBR concentration-dependent manner in PTZ–seizure larvae (Figure 5C). In these tests, a BBR effect that occurred at 75 μM was shown to be nearly similar to that seen with VPA at 120 μM. These results suggest that BBR probably has a therapeutic effect on PTZ-induced seizures in zebrafish. Therefore, we speculate that BBR might be able to suppress an epilepsy-like seizure by upregulating STX1B expression and also that the level of STX1B is associated with seizure outlook.

### STX1B Mediated the Therapeutic Effect of Berberine on PTZ-Induced Seizure in Zebrafish

**FIGURE 6:**
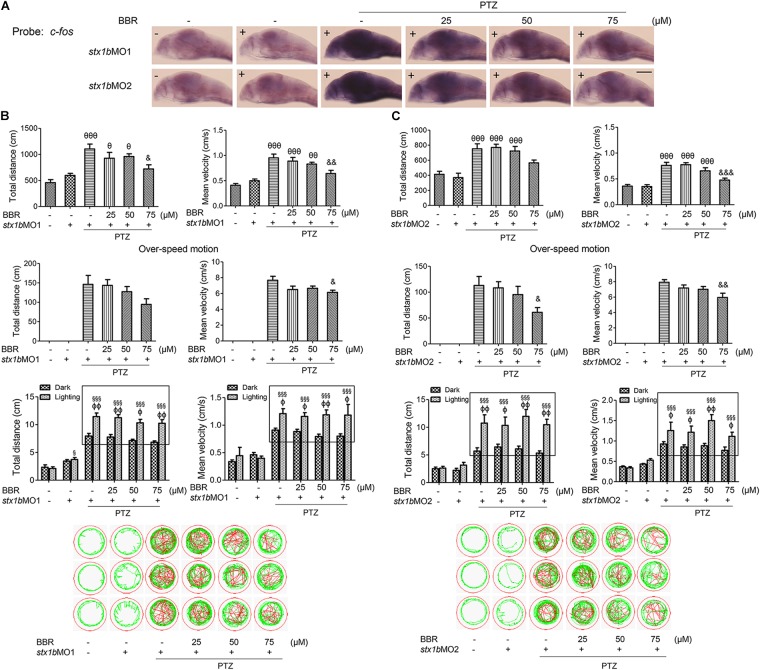
Downregulation of STX1B weakened the effects of BBR on anticonvulsant in the PTZ-induced seizure zebrafish model. **(A)** Hybridization *in situ* showed that there was a change in the *c-fos* messenger RNA level in the larval (7 dpf) brain that was induced by BBR in the PTZ plus *stx1b* morpholino oligos group versus in the three control groups of wild-type, *stx1b* morpholino oligos injection, and PTZ plus *stx1b* morpholino oligos (*n* = 20). **(B,C)** STX1B downregulation attenuated the efficiency of BBR inhibition on larval overexcited locomotion in terms of speed and distance under non-stimulation conditions and eliminated the action of BBR under dark–light transitions. Swimming trajectories are presented in 2 min recording charts; red tracks indicate over locomotion, while the rectangles indicate the difference between light–dark transitions (*n* = 24). ^&^*P* < 0.05, ^&&^*P* < 0.01, and ^&&&^*P* < 0.001 vs. PTZ plus *stx1b* morpholino oligos model; ^𝜃^*P* < 0.05, ^𝜃𝜃^*P* < 0.01, and ^𝜃𝜃𝜃^*P* < 0.001 vs. *stx1b* morpholino oligos model; ^§^*P* < 0.05 and ^§§§^*P* < 0.001 vs. wild-type in the light condition; ^Φ^*P* < 0.01 and ^ΦΦ^*P* < 0.01 indicates light vs. dark.

**FIGURE 7:**
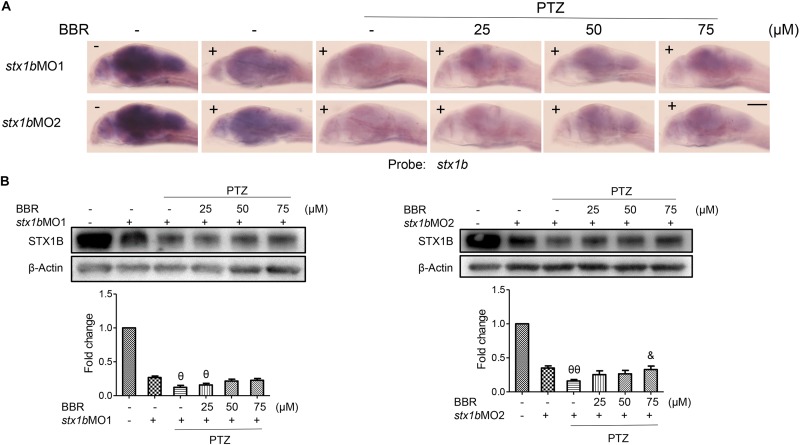
*Stx1b* morpholino oligos injection suppressed BBR activation on STX1B expression in the PTZ-model larvae. **(A)** Hybridization *in situ* results show that a change of the *stx1b* messenger RNA level in the larval (7 dpf) brain was induced by BBR with *stx1b* morpholino oligos injection in the PTZ-model zebrafish, as compared with in the wild-type, *stx1b* morpholino oligos injection, and PTZ plus *stx1b* morpholino oligos groups (*n* = 20). **(B)** Western blotting results indicated a change of the STX1B protein level similar to the change of the *stx1b* messenger RNA level under the same treatments (*n* = 3). ^&^*P* < 0.05 vs. PTZ plus *stx1b* morpholino oligos model; ^𝜃^*P* < 0.05 and ^𝜃𝜃^*P* < 0.01 vs. *stx1b* morpholino oligos model.

**FIGURE 8:**
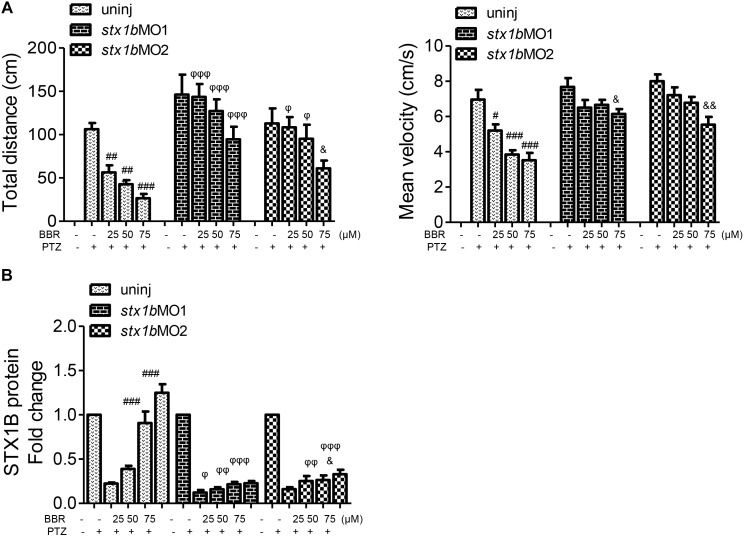
Comparative analysis of epilepsy-like seizure and STX1B protein levels between BBR with and without *stx1b* morpholino oligos injection in the PTZ-induced seizure zebrafish. **(A)** Behavioral comparison indicates that the BBR effect of antiseizure was weakened by *stx1b* morpholino oligo injection. **(B)** Comparison of STX1B protein levels induced by BBR between *stx1b* gene knockdown and non-knockdown. Western blotting showed that levels of STX1B protein were significantly decreased by *stx1b* morpholino oligo injection under BBR existence. The histograms are generated from data in Figure 5 of behavior and western blotting, Figure 6 of behavior, and Figure 7 of western blotting. ^#^*P* < 0.05, ^##^*P* < 0.01, and ^###^*P* < 0.001 vs. PTZ model; ^&^*P* < 0.05 and ^&&^*P* < 0.01 vs. PTZ plus *stx1b* morpholino oligos model. ^φ^ *P* < 0.05, ^φ^ ^φ^ *P* < 0.01, and ^φ^ ^φ^ ^φ^ *P* < 0.001 indicate differences in the comparison between uninjected and morpholino oligo injection in the PTZ model under the same concentration of BBR, respectively.

Since BBR is likely to suppress the onset of PTZ-induced seizures in zebrafish accompanying the enhancement of STX1B expression, we evaluated whether or not BBR was dependent on STX1B protein to play the anticonvulsant role in the zebrafish seizure model. *In situ* hybridization results showed that, under the *stx1b* morpholino oligos injection condition, BBR only moderately reduced the *c-fos* level in the brain region of PTZ-treated zebrafish (Figure 6A). Behavioral results revealed that BBR mildly attenuated the increase of the average velocity and total movement distance including the abnormal trajectory and overspeed (clonus-like convulsions) in the group of PTZ plus *stx1b* morpholino oligos in the dark condition (Figures 6B,C), suggesting that *stx1b* knockdown caused BBR inhibition action that was obviously weaker than that in PTZ-only-treated zebrafish (Figure 5A). Moreover, BBR did not prevent an overexcited response in the light stimulation condition (Figures 6B,C). Subsequently, we studied the efficiency of BBR activating STX1B expression under *stx1b* knockdown in the PTZ-treated larvae and found that BBR only slightly raised *stx1b* messenger RNA and protein levels in the PTZ plus *stx1b* morpholino oligos group, in which the STX1B level was lower than that in the *stx1b* morpholino oligos group and considerably lower than that in the normal control group (Figures 7A,B). Furthermore, a data comparative analysis was carried out between BBR with and without *stx1b* morpholino oligos injection and indicated that STX1B downregulation significantly weakened or even eliminated BBR efficiency for suppressing an epileptic seizure including abnormal trajectory and overspeed in the dark condition and STX1B protein levels (Figure 8) as well as photosensitive seizures (Figure 6) of the PTZ-induced seizure in zebrafish. Considering the cohesive tendency between the STX1B level variation and the larval behavior results, we infer that STX1B is an important mediator for BBR action on anticonvulsants, in particular for the inhibition of photosensitive seizures that may require proper STX1B expression.

## Discussion

STX1B is a synapse fusion protein that is associated with the release of neurotransmitters, and mutations of the *stx1b* gene lead to familial fever-associated epilepsy syndromes in humans (Sudhof, 2013; Schubert et al., 2014). *Stx1b* knockout mice (Stx1b^-/-^) demonstrated damaged glutamatergic and GABAergic synaptic transmissions (Mishima et al., 2014), while Stx1b^+/-^ mice exhibited a reduced release of GABA and a disturbance of the dopaminergic system in the central nervous system (Fujiwara et al., 2017). GABA is an important inhibitory neurotransmitter in the brain, and the roles of GABA and its receptor in epilepsy have been widely studied (Ferando and Mody, 2012). PTZ is a regular compound used to trigger seizures in animal models that selectively blocks GABA receptor channels and weakens GABA-mediated neurotransmitter systems, causing the neurons to overexcite (Soares et al., 2017). In this study, we used PTZ to establish a zebrafish seizure model and researched STX1B functions in epilepsy-like seizures, including photosensitive seizures. We found that the *stx1b* gene expression decrease that accompanies epilepsy-like seizure aggravation, was induced by PTZ, and that STX1B increase and the alleviation of a seizure were observed under treatment of the anti-epilepsy drug VPA. Moreover, *stx1b* knockdown made zebrafish more sensitive to PTZ than just PTZ treatment did (Figure 3). This indicates that STX1B decline is closely related with PTZ-induced epileptic seizures and that STX1B might be a protein marker in a PTZ-induced seizure model for the screening of anticonvulsant drugs. The alignment of human and zebrafish STX1B protein sequences showed that the STX1B proteins have a high homology of 98% (Figure 2A) and that they possess the same structural domain as compared with syntaxin and SNARE^2^. Altogether, these results hint that STX1B may exert similar biological functions in zebrafish as in humans.

A photosensitive seizure is a kind of epileptic response to the visual stimuli of color and light. Triggers can include television and computer games, among many others (Martins da Silva and Leal, 2017). At present, the relationship between photosensitive epilepsy and other genes involved is not very clear, in spite of bromodomain-containing protein 2 and CHD2 being known as a likely susceptible gene in photosensitive epilepsy (Lorenz et al., 2006; Galizia et al., 2015; Poleon and Szaflarski, 2017). However, the correlation of STX1B to photosensitive epileptic seizures has not been reported until now. Photosensitive epilepsy does not only occur in a single kind of epilepsy syndrome; it has also been found in juvenile myoclonic epilepsy, eyelid myoclonia (Jeavons syndrome), and Dravet syndrome (Poleon and Szaflarski, 2017). According to a study, photosensitivity was reported to occur in approximately 31% of those with juvenile myoclonic epilepsy (Wolf and Goosses, 1986). Photosensitive epilepsy usually occurs in adolescents: it is estimated that patients between the ages of 7 and 19 years are about five times more likely than those in other age groups to demonstrate the condition (de Bittencourt, 2004). Therefore, it could be argued that photosensitive epilepsy is a serious threat to the physical and mental health of teenagers. In this study, we explore the correlation between STX1B level and photosensitive seizures under the condition of a dark–light shift in a PTZ–seizure zebrafish model. Our behavioral experiments show that PTZ treatment with *stx1b* knockdown made the larvae oversensitive to light stimulation, and the *c-fos* level (*c-fos* is recognized as a marker for neuronal activity, and the expression level of *c-fos* is positively correlated with the degree of epileptic seizure) (Baraban et al., 2005) in the zebrafish brain was significantly higher in the PTZ-treated zebrafish with *stx1b* knockdown than in the PTZ-only model group. In contrast, STX1B overexpression decreased larval overspeed swimming behaviors under light stimuli and suppressed the *c-fos* expression in the zebrafish brain, as compared with in the PTZ group. Therefore, we suppose that the STX1B protein can alleviate PTZ-induced photosensitive seizures.

Despite there being no known reports of STX1B correlating with photosensitivity, several studies have implicated CHD2 in photosensitivity and have shown that CHD2 mutation is the first identified cause of the archetypal generalized photosensitive epilepsy syndrome, with CHD2 knockdown markedly increased in the case of zebrafish larval photosensitivity (Galizia et al., 2015). According to other reports, *chd2* gene mutations were described in MAE (Carvill et al., 2013; Thomas et al., 2015); at the same time, *stx1b* gene variants or deletions can also be involved in the etiology of MAE (Vlaskamp et al., 2016). Since both *stx1b* and *chd2* gene mutations can lead to MAE, whether or not STX1B is also related to photosensitivity epilepsy like CHD2 is, remains a question. MAE is an epilepsy characterized by the occurrence of myoclonic–atonic seizures, while myoclonic seizures are a typical symptom in the PTZ-induced seizure model (Frye and Muscatiello, 2001; Poplawska et al., 2015). In association with our results, these studies imply that STX1B may be associated with photosensitive epilepsy. However, the relationship between STX1B and photosensitive response still needs further clinical study.

Berberine was reported to have a protection effect on neurodegenerative and neuropsychiatric disorders with respect to its antioxidant and anti-inflammatory roles (Yoo et al., 2006, 2008; Sedaghat et al., 2017). Some studies have shown that BBR antagonized *N*-methyl-D-aspartate-induced excitotoxicity in gerbil hippocampal neurons (Yoo et al., 2008) and inhibited morphine-induced locomotor sensitization in mice (Yoo et al., 2006). Moreover, BBR attenuated a repeated nicotine-induced behavioral sensitization by decreasing postsynaptic neuronal activation in rats (Lee et al., 2007). These findings suggest that BBR probably is involved in the inhibition of neuron-locomotion overactivity, but published reports about the action of BBR in epilepsy remain scarce. In the present study, we found that BBR alleviated the overexcitation reaction and decreased the level of *c-fos* induced by PTZ, yet rescued the level of *stx1b* transcription suppressed by PTZ. When STX1B was downregulated, BBR’s therapeutic effect on a photosensitive seizure was significantly reduced or eliminated, suggesting that BBR’s inhibitory effect on a photosensitive seizure was dependent on the presence of STX1B protein. We speculate that BBR may indirectly activate some transcription factors to enhance the expression of the *stx1b* gene.

In summary, PTZ induces an epilepsy-like seizure, including photosensitive seizures in zebrafish, which may be partially mediated by STX1B deficiency. Adequate STX1B levels can slow down the hyperexcitation locomotion induced by PTZ in zebrafish. BBR can suppress PTZ-induced seizures in zebrafish by raising STX1B levels. Further research on the relationship between STX1B, BBR, and seizures may provide new clues for the development of novel antiepileptic drugs.

## Author Contributions

J-PZ conceived and designed the project. Y-MZ and BC performed the experiments and treated the data. J-DJ provided substantial discussion for writing the manuscript. Y-MZ and J-PZ wrote the manuscript.

## Conflict of Interest Statement

The authors declare that the research was conducted in the absence of any commercial or financial relationships that could be construed as a potential conflict of interest.

## Footnotes

1 https://store.gene-tools.com/node/333

2 https://blast.ncbi.nlm.nih.gov/Blast.cgi#alnHdr_66393091
