# Supplementary figures and images for: Syntaxin 1B Mediates Berberine’s Roles in Epilepsy-Like Behavior in a Pentylenetetrazole-Induced Seizure Zebrafish Model
Source: Front Mol Neurosci. 2018 Nov 26;11:378. doi: 10.3389/fnmol.2018.00378 (PMC6275243; doi:10.3389/fnmol.2018.00378)

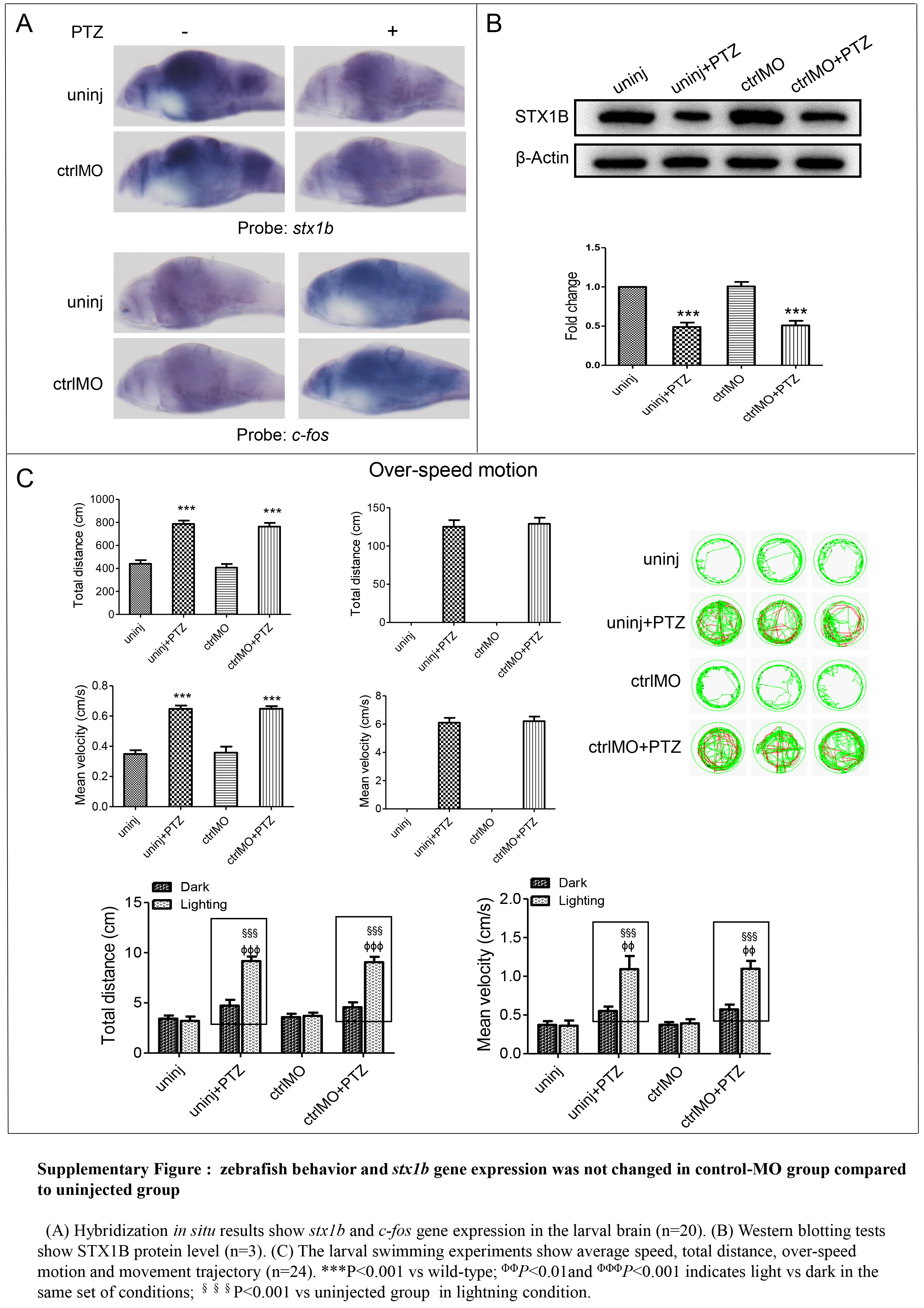

Supplement: Supplementary file 1 [file Image_1.JPEG]
